# Comprehensive Analysis of 34 Edible Flowers by the Determination of Nutritional Composition and Antioxidant Capacity Planted in Yunnan Province China

**DOI:** 10.3390/molecules28135260

**Published:** 2023-07-06

**Authors:** Xing-Kai Zhang, Guan-Hua Cao, Yue Bi, Xiao-Hai Liu, Hong-Mei Yin, Jia-Fang Zuo, Wen Xu, Hong-Dong Li, Sen He, Xu-Hong Zhou

**Affiliations:** 1 Yunnan Key Laboratory of Southern Medicine Utilization, School of Chinese Materia Medica, Yunnan University of Chinese Medicine, Kunming 650500, China; zxkaky@outlook.com (X.-K.Z.); 10004@ynutcm.edu.cn (G.-H.C.);; 2 Office of Science and Technology, Yunnan University of Chinese Medicine, Kunming 650500, China

**Keywords:** edible flowers, Asteraceae flowers, Rosaceae flowers, Gentianaceae flowers, nutrient composition, antioxidant capacity

## Abstract

The main purpose of this study was to reveal the nutritional value and antioxidant activity of 34 edible flowers that grew in Yunnan Province, China, through a comprehensive assessment of their nutritional composition and antioxidant indices. The results showed that sample A3 of Asteraceae flowers had the highest total flavonoid content, with a value of 8.53%, and the maximum contents of vitamin C and reducing sugars were from Rosaceae sample R1 and Gentianaceae sample G3, with values of 143.80 mg/100 g and 7.82%, respectively. Samples R2 and R3 of Rosaceae were the top two flowers in terms of comprehensive nutritional quality. In addition, the antioxidant capacity of Rosaceae samples was evidently better than that of three others, in which Sample R1 had the maximum values in hydroxyl radical (^·^OH) scavenging and superoxide anion radical (^·^O_2_^−^) scavenging rates, and samples R2 and R3 showed a high total antioxidant capacity and 2,2-diphenyl-1-pyridylhydrazine (DPPH) scavenging rate, respectively. Taken together, there were significant differences in the nutrient contents and antioxidant properties of these 34 flowers, and the comprehensive quality of Rosaceae samples was generally better than the other three families. This study provides references for 34 edible flowers to be used as dietary supplements and important sources of natural antioxidants.

## 1. Introduction

Flowers are the reproductive structures of flowering plants and could be classed as edible and non-edible flowers based on whether they have deleterious effects on human health when consumed [1]. Many flowers with a long history of edibility mainly depend on their inherent nutritional and medicinal properties [2,3]. The consumption of edible flowers can be traced back to China, ancient Greece, Europe, the Middle East and Japan [4]. Especially in China, edible flowers have been used as herbs, spices, food ingredients and decorative food garnishes since ancient times [5,6]. Edible flowers are often seen as common foods that are similar to vegetables and fruits, but there are no recommended daily amounts. At present, most studies on edible flowers concentrate on the substances and essential oils that produce the source of floral aroma and few studies focus on the intrinsic nutritional value and material composition [7]. In recent years, with the advancement of people’s living standards and the pursuit of a healthy lifestyle, the positive health effects of edible flowers on the human body, as well as their nutritional value, are gradually attracting the interest of researchers [3]. The discovery of new edible flowers provided materials for the development of functional food.

Ferric reducing antioxidant power (FRAP), 2,2-diphenyl-1-picrylhydrazyl (DPPH) and free radical (hydroxyl radical OH^.^ and superoxide anion ^·^O_2_^−^) scavenging rates are commonly used as parameters to evaluate antioxidant capacity. These substances play an important role in the physiological processes and pathogenesis of many diseases [8]; for example, an excess of reactive oxygen species in the human body will lead to toxicity and trigger a series of physiological and biochemical reactions that will cause cellular damage to the organism’s health [9]. Many ornamental and edible flowers are reported to be vital sources of natural antioxidants, which can inhibit most of the negative effects of free radicals on the body, e.g., cancer, cardiovascular disease, chronic inflammation and aging [10,11,12]. However, a majority of antioxidants used in food or beauty products today are synthetic antioxidants that may cause certain diseases if used for an extended period of time [8]. Thus, safe and effective antioxidants from natural and harmless products, e.g., edible flowers, would be in large demand.

Although edible flowers have received increasing attention from scholars due to their special nutritional value, most studies on edible flowers have so far focused on the determination of bioactive substances of certain flowers, while neglecting the determination of the composition and contents of nutrients that can be used as food in their own right. In addition, no systematic comparison of the antioxidant properties of a large number of edible flowers has been reported, particularly for different families. Therefore, it is critical to research the nutrient composition, nutrient content and antioxidant properties of edible flowers in order to better understand their health benefits in humans. For these reasons, the aim of this study was to determine the nutrient content and antioxidant properties of 34 flowers from four plant families planted in Yunnan Province, China, with a goal of providing data reference for future development and utilization.

## 2. Results

### 2.1. Determination Results of Nutrient Contents

The main nutrient contents of these 34 flowers are listed in Table 1, including moisture, total flavonoids, vitamin C, total soluble sugars, reducing sugars, ash and total proteins. The moisture contents of 34 flower samples varied from 71.83 to 90.26%, in which sample A8 from the family Asteraceae had the largest value, while sample R3 from the family Rosaceae possessed the lowest moisture content. Total flavonoids are the main active substances in flowers, with a strong antioxidative effect that can effectively remove oxygen free radicals to protect the body from oxidative damage [13]. There were significant differences in the total flavonoid contents of these 34 flowers, and the highest content of 8.53% appeared in sample A3 of the family Asteraceae. In contrast, sample C6 from the family Caryophyllaceae had the lowest content of total flavonoids, only 0.35%. Vitamin C is another important natural antioxidant in flowers, and the vitamin C contents of these 34 flowers ranged from 1.62 to 143.80 mg/100 g, which indicated that the content of Rosaceae sample R1 was more than 88 times that of Asteraceae flower sample A4. The contents of total soluble sugars greatly affect the taste of edible flowers, consisting of monosaccharides, disaccharides and polysaccharides. Significant differences were observed among these 34 flowers, with a minimum value of 3.78% from sample R6 and a maximum value of 112.60% from sample R9. In addition, the amounts of reducing sugars, ash and total proteins were also determined, with ranges of 0.04~7.82%, 3.55~9.86% and 2.79~25.43%, respectively.

### 2.2. Results of Antioxidant Capacity

#### 2.2.1. Total Antioxidant Activity

**Figure 1:**
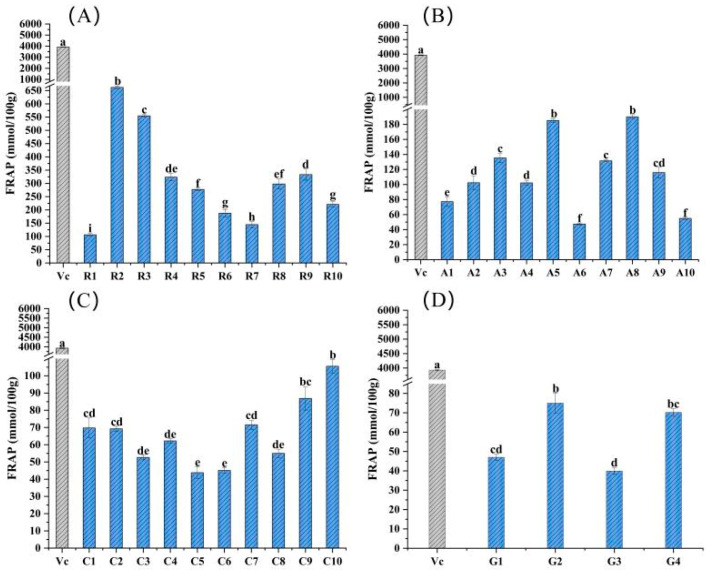
Total antioxidant capacity assessment of the 34 edible flower samples, using the parameter of FRAP. (**A**–**D**) Total antioxidant capacity of flower samples from families Rosaceae, Asteraceae, Caryophyllaceae and Gentianaceae, respectively. Vitamin C was a positive control. Different lowercase letters indicate statistically significant differences in the FRAP values of flower samples within the same family, *p* < 0.05.

The total antioxidant activities of these 34 flower samples and vitamin C (positive control) were evaluated by the FRAP assay. As shown in Figure 1, Rosaceae flower samples generally had higher antioxidant properties, with the range from 106.33 to 662.20 mmol/100 g fresh weight (FW), and the antioxidant capacity of sample R2 was significantly greater than that of others (*p* < 0.05) but obviously lower than the 3928 mmol/100 g of vitamin C (Figure 1A). However, there was no obvious difference in the total antioxidant activity of samples from the families Asteraceae, Caryophyllaceae and Gentianaceae. Samples of A5 and A8 had a relatively high antioxidant capacity compared to others in the same family of Asteraceae, and the FRAP values were 185.00 and 190.00 mmol/100 g FW, respectively (Figure 1B). For the family Caryophyllaceae, sample C10 had the highest total antioxidant capacity, with a FRAP value of 106.00 mmol/100 g FW, which was significantly higher than others (Figure 1C). The total antioxidant capacity of sample G2 among Gentianaceae flowers was the largest, with a FRAP value of 75.00 mmol/100 g FW, making it significantly (*p* < 0.05) different from other flower samples from the same family (Figure 1D).

#### 2.2.2. DPPH Scavenging Ability

**Figure 2:**
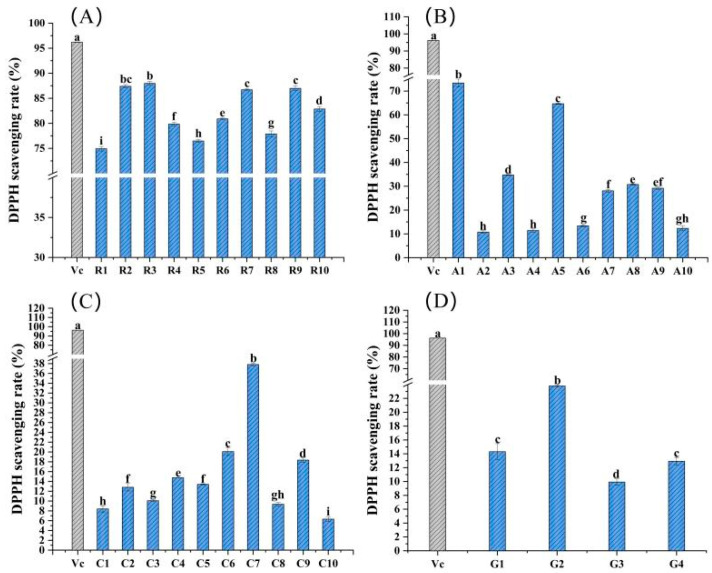
DPPH scavenging rates of the 34 edible flower samples. (**A**–**D**) DPPH scavenging rates of flower samples from the families Rosaceae, Asteraceae, Caryophyllaceae and Gentianaceae, respectively. Vitamin C was a positive control. Different lowercase letters indicate statistically significant differences in the values of flower samples within the same family, *p* < 0.05.

The DPPH scavenging ability of 34 edible flowers is shown in Figure 2, with vitamin C serving as a positive control. Sample R3 had the highest DPPH scavenging rate in Rosaceae flowers, and the scavenging rate reached 87.98% with a sample addition volume of 10 mg/mL, just less than 96.22% of vitamin C, which was significantly different from other flower samples in the same family (*p* < 0.05) (Figure 2A). Sample A1 of family Asteraceae had the highest DPPH scavenging rate, i.e., 73.36%, which was significantly higher than that of others, followed by A5 and A3 (Figure 2B). In addition, sample C7 of Caryophyllaceae and sample G2 of Gentianaceae showed a strong DPPH scavenging ability in their respective families, and the reference values were 37.82 and 23.79%, respectively (Figure 2C,D).

#### 2.2.3. OH Scavenging Ability

**Figure 3:**
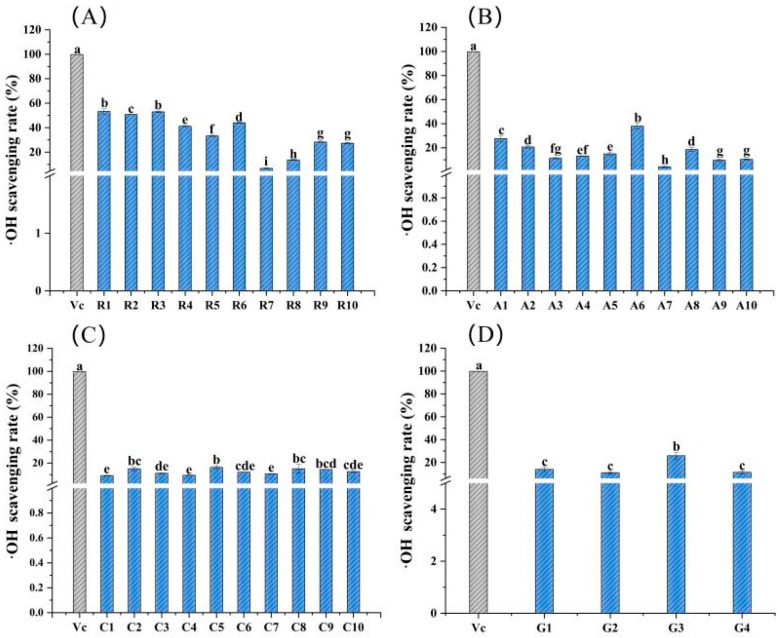
^·^OH scavenging rates of the 34 edible flower samples. (**A**–**D**) ^·^OH scavenging rates of flower samples from the families Rosaceae, Asteraceae, Caryophyllaceae and Gentianaceae, respectively. Vitamin C was a positive control. Different lowercase letters indicate statistically significant differences in the values of flower samples within the same family, *p* < 0.05.

Figure 3 depicts the ^·^OH scavenging rates of 34 edible flowers and a positive control (vitamin C). As determined by the comprehensive comparison, the ^·^OH scavenging ability of sample R1 from the family Rosaceae was the highest, with a value of 53.26%, followed by 52.97% for R3 and 50.85% for R2. In fact, the ^·^OH scavenging rates of Rosaceae flower samples were generally better than those of the other three families at the same mass concentration, and the range was 6.74 to 53.26% (Figure 3A). Among the flowers of Asteraceae, the ^·^OH scavenging rates are sorted as follows: A6 > A1 > A8 > A2 > A4 > A5 > A3 > A9 > A10 > A7 (Figure 3B). For 10 samples of the family Caryophyllaceae, the ^·^OH scavenging ability was fairly close and less than 16.28% (Figure 3C). The ^·^OH scavenging ability of four flower samples from Gentianaceae was higher than 11.04%, and the highest value was 25.89%, which belonged to sample G3 (Figure 3D).

#### 2.2.4. ^·^O_2_^−^ Scavenging Ability

**Figure 4:**
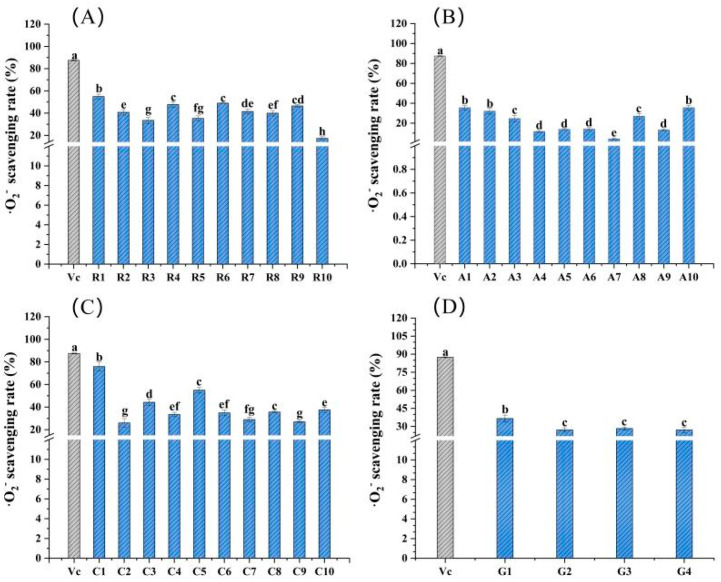
^·^O_2_^−^ scavenging rates of the 34 edible flower samples. (**A**–**D**) ^·^O_2_^−^ scavenging rates of flower samples from family Rosaceae, Asteraceae, Caryophyllaceae and Gentianaceae, respectively. Vitamin C was a positive control. Different lowercase letters indicate statistically significant differences in the values of flower samples within the same family, *p* < 0.05.

The ^^·^^O_2_^−^ scavenging rates of 34 flower samples and vitamin C are shown in Figure 4. In general, the ^·^O_2_^−^ scavenging ability of Caryophyllaceae sample C1 was the largest, with a value of 75.83%, close to the 87.47% of vitamin C, followed by 54.95% for Caryophyllaceae sample C5 and 54.93% for Rosaceae sample R1. For the 10 samples of Rosaceae, sample R1 had the highest ^·^O_2_^−^ scavenging rate, i.e., 54.93%, and was significantly greater than others in the same family (*p* < 0.05) (Figure 4A). Among 10 flower samples of family Asteraceae, the ^·^O_2_^−^ scavenging rates of samples A1 and A10 were significantly higher than others (*p* < 0.05); both of the values were about 35.56% (Figure 4B). For the family Caryophyllaceae, sample C1 had the largest ^·^O_2_^−^ scavenging rate, i.e., 75.83%, and was significantly higher than that of others (*p* < 0.05) (Figure 4C). As for Gentianaceae flower samples, the ^·^O_2_^−^ scavenging ability of sample G1 was significantly stronger than that of the other three flower samples (*p* < 0.05). However, there was no significant difference among samples G2, G3 and G4 (*p* > 0.05) (Figure 4D).

### 2.3. Comprehensive Evaluation Based on Flower Nutrient Value and Antioxidant Capacity

#### 2.3.1. Correlation Analysis

**Figure 5:**
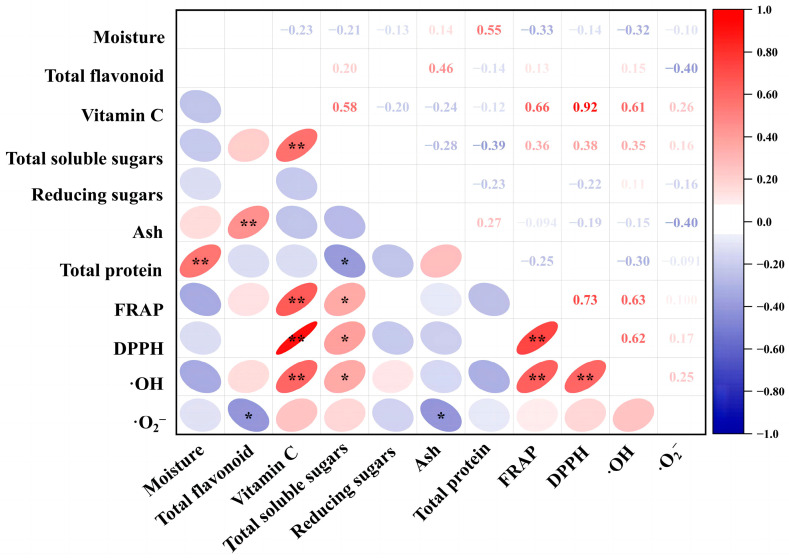
Pearson correlation analysis of nutrient and antioxidant indices of the 34 edible flower samples. Ellipse in color indicates the correlation between 2 indicators that distributed on the vertical and horizontal axes, respectively. The transition of shape and color of ellipse from round to flat and from blue to red indicates an increase in the correlation; * and ** denote significant correlation at the levels of *p* < 0.05 and *p* < 0.01, respectively.

Pearson’s correlation analysis is frequently utilized for estimating the degree of correlation between two parameters. As shown in Figure 5, the sample correlation result showed that vitamin C content was significantly (*p* < 0.05) positively correlated with the FRAP value, DPPH scavenging rate and ^·^OH scavenging rate, but particularly the DPPH scavenging rate, with a correlation coefficient of 0.92. In addition, any two of the three parameters, FRAP value, DPPH scavenging rate and ^·^OH scavenging rate, showed a highly significant positive correlation.

#### 2.3.2. Principal Component Analysis and Cluster Analysis

**Figure 6:**
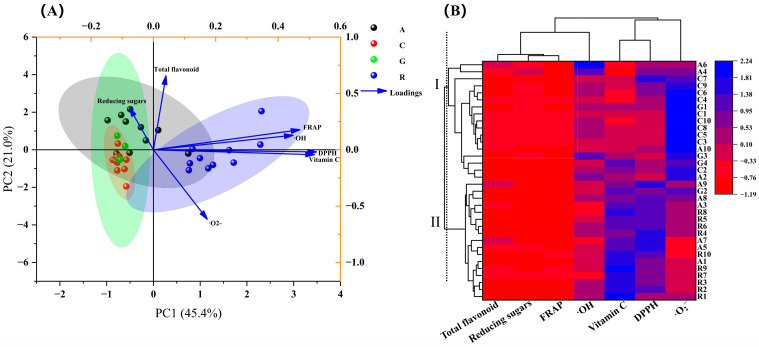
Principal component analysis (**A**) and cluster analysis (**B**) of the comprehensive quality of the 34 edible flower samples. In (**A**), 4 corresponding colors for A, C, G and R represent flower samples of Asteraceae, Caryophyllaceae, Gentianaceae and Rosaceae, respectively. (**B**) shows the cluster analysis conducted using the parameter of “Manhattan distance 2.5” in the tool of “heat map with Dendrogram” provided by the software Origin 2021. These 34 edible flower samples were classified into 2 major categories, Ⅰ and II.

Based on the results of the nutrient composition, antioxidant capacity and the correlation analysis, seven indices of total flavonoids, vitamin C, reducing sugars, FRAP values and free radical scavenging rates of DPPH, ^·^OH and ^·^O_2_^−^, that influenced the sample quality were selected for the principal component analysis, and a classification model of the nutritional value and antioxidant capacity of 34 edible flowers was constructed, as shown in Figure 6A. The variance contributions of principal component 1 (PC1), principal component 2 (PC2) and principal component 3 (PC3) were 45.446, 20.981, and 14.844%, respectively, and the cumulative variance contributions of the three principal components reached 81.27%. The PC1-reflected indicators were vitamin C content, FRAP value and the scavenging rates of DPPH and ^·^OH. The total flavonoids and reducing sugar contents were the major parameters that reflected PC2. PC3 was mainly determined by reducing sugar contents. The PCA results showed that there was no significant separation in nutritional quality and antioxidant capacity among the four families of flower samples (Figure 6A). The classification model based on the cluster-analysis results showed that the 34 edible flower samples were classified into two major categories, the first of which contained 17 samples, namely A6, A4, C7, C9, C4, G1, C1, C10, C8, C5, C3, A10, G3, G4, C2 and A2, and the remaining 17 samples were clustered as category II (Figure 6B).

### 2.4. Comprehensive Quality of 34 Edible Flowers Based on PCA Models

In order to compare the quality of the 34 flower samples more accurately, three principal components were extracted based on the results of principal component analysis with eigenvalues greater than 1 as the extraction criterion, of which the cumulative variance contribution was more than 80% (Table 2). This indicated that these three principal components can capture the majority of the information in the samples [14]. As a result, the three extracted principal components can be used instead of the seven indices to assess the comprehensive quality of the 34 flower samples. The principal component score for each sample was subsequently calculated using SPSS 26.0 software based on the standardized data and were indicated as F1, F2 and F3, respectively. The variance contribution of each component was weighted to determine the comprehensive score, which was then determined as follows (Formula (1)):

(1) $$C_{q}=F1\times Z1+F2\times Z2+F3\times Z3$$

where C_q_ signifies the total score; F1, F2 and F3 denote the respective scores of the three principal components; and Z1, Z2 and Z3 indicate the weighted variance contribution of each of the three extracted principal components, respectively.

The scores of the three principal components of the 34 edible flower samples, as well as the weighted comprehensive scores and ranks, are listed in Table 3. The results showed that the comprehensive quality of Rosaceae samples was generally better than that of the other three families, and the nine samples of the Rosaceae family were ranked in the top nine. Among the 34 flower samples, sample R2 had the highest comprehensive score and the best quality, followed by sample R3, which came in second, but sample C4 had the lowest comprehensive score and the worst nutritional and antioxidant quality.

## 3. Materials and Methods

### 3.1. Flower Sample Collection

**Figure 7:**
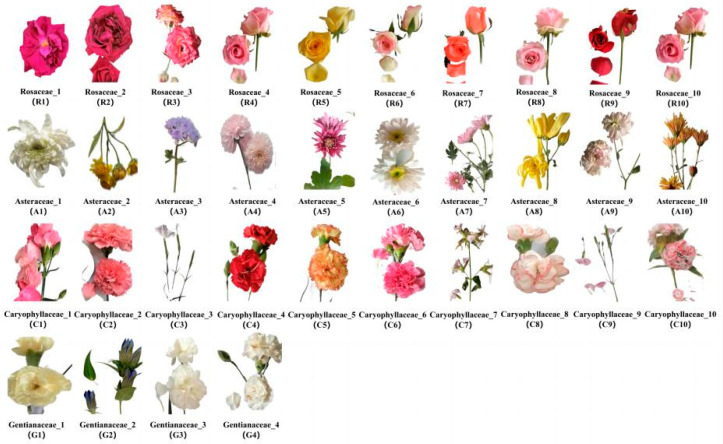
The 34 edible flower samples used in this study.

In total, 34 edible flower species of 4 families, namely Rosaceae, Asteraceae, Caryophyllaceae and Gentianaceae, were collected from Professor Xu-hong Zhou’s plantation (Figure 7), located in Jinning District of Kunming, Yunnan Province. The identification of the families for these 34 flowers mainly depended on the morphological and structural characteristics of flowers, and the identification was conducted by Professor Xu-hong Zhou, who is an expert in the field of plant taxonomy. Flowers of Rosaceae are bisexual flowers, generally have 5 petals with bright colors that arrange themselves in complex flower patterns, e.g., double petals and single petals. The central part of the flowers has an obvious disk that is round or saucer-shaped, with the stamen and pistil distributed on it. The perianth and stamen combine to form a flower tube. The most important characteristic of Asteraceae flowers is that the inflorescence is capitulum, mainly including tongue and tube flower. Flowers of Caryophyllaceae are bisexual flowers that are either solitary or arranged in cymes, and each flower has 4~5 petals that often are unguiculate. Gentianaceae flowers are mainly bisexual flowers that are often blue and arranged in cymes. There are 4~5 cleft in the calyx and corolla of Gentianaceae flowers, and the stamens attach to the corolla.

### 3.2. Reagents and Apparatus

Sodium hydroxide, potassium dichromate, formaldehyde, copper sulfate, o-benzene-triol, sodium phosphate and potassium ferricyanide were purchased from Tianjin Fengchuan Chemical Reagent Technology Co., Ltd. (Tianjin, China). Sodium thiosulfate, potassium iodide, potassium sulfate, ferrous sulfate, ammonium acetate and soluble starch were purchased from Guangdong Guanghua Sci-Tech Co., Ltd. (Guangdong, China). All reagents were of analytical purity level. The instruments and equipment used in the experiments included an ultraviolet–visible spectrophotometer (Shimadzu UV-2550, Shanghai, China), crude fat tester (SZF-06A, Shanghai, China), ultrasonic cleaner (SK72010HP, Shanghai, China), rotary evaporator (Aika HB10 SO96, Guangzhou, China) and microwave digestion instrument (MWD-800, Shanghai, China).

### 3.3. Nutritional Composition Analysis

The nutritional composition analysis of the flower samples included the contents of moisture, total flavonoids, vitamin C (L-ascorbic acid), total soluble sugars, reducing sugars, ash and total proteins.

#### 3.3.1. Laboratory Analysis

The proximate compositions of moisture, ash and total proteins were determined according to the methods described in the Chinese National Standards of GB 5009.3-2016, GB 5009.4-2016 and GB 5009.5-2016, respectively. The contents of total soluble sugars and reducing sugars were determined using the methods of anthrone colorimetric [15] and 3, 5-dinitro salicylic acid colorimetric [16], respectively. Vitamin C contents were determined by the method of iodometric method reported by Dumbravă [17]. All the determinations were made in 3 replicates.

#### 3.3.2. Determination of the Total Flavonoid Contents

The total flavonoids of flowers were extracted first and then were determined using the method reported by Wu, with minor modifications [18]. The protocol of total flavonoids extraction from samples are as follows. The dried sample powder (1.000 g) in a round-bottom flask was added to 40 mL of 80% ethanol for reflux extraction for 1 h, and the supernatant was collected by filtration. Filtrate residue was used to reflux extraction again with the same condition. The combined filtrate from the filtration was adjusted to 100 mL, using 80% ethanol. The standard curve was measured at 400 nm with rutin as a standard, of which the linear equation of standard curve is y = 15.58x − 0.0186 (R^2^ = 0.999). The total flavonoid contents of samples were calculated using the linear equation.

### 3.4. Determination of Antioxidant Capacity

The antioxidant capacity of flower samples was estimated using the parameters of FRAP and free radical scavenging rates of DPPH, ^·^OH and ^·^O_2_^−^.

#### 3.4.1. Determination of Total Antioxidant Activity

FRAP assay was employed to evaluate the total antioxidant activity of flowers, and the method used was reported by Benzie [19], with slight modifications. First, 1 mL of sample solution was mixed with 4 mL of 70% ethanol, and then 0.2 mL of mixed solution consisting of 1 mL sample solution and 4 mL 70% ethanol was taken to react with 6 mL of the FRAP reagent at 37 °C for 20 min, and the absorbance was determined at 593 nm. The standard curve was created under the same operating conditions by replacing the sample solution with FeSO_4_ standard solution. The regression equation of y = 0.119x − 0.0196 (R^2^ = 0.992) was generated for the calculation of total antioxidant activity. The assay for each sample was conducted in triplicate.

#### 3.4.2. Determination of DPPH Scavenging Ability

The DPPH scavenging power was determined by referring to the report of Stefaniak [20], with minor modifications. The DPPH reagent (0.1 μmol/L) was prepared by dissolving 4 mg DPPH in 100 mL of 70% ethanol, and then 4 mL of 0.1 μmol/L DPPH regent was mixed with 0.2 mL of sample solution for chemical reaction. The reaction mixture was fully protected in the dark, at 37 °C, for 20 min. The absorbance (A_1_) was measured at 517 nm. Absorbance values of A_2_ and A_0_ were determined by using 4/0.2 mL of 70% ethanol instead of DPPH solution or sample solution in the reactive system, respectively. Reduced vitamin C was used as a positive control. The determinations assay for each sample was conducted in triplicate. The DPPH scavenging rates for samples were calculated using the Formula (2):

(2) $$DPPH\;scavenging\;rate\;\left(\%\right)=\left(1-\frac{A_{1}-A_{2}}{A_{0}}\right)\times 100\%$$

#### 3.4.3. Determination of ^·^OH Scavenging Ability

The ability of samples to inhibit ^·^OH was evaluated using the Fenton method [21], with slight modifications. A mixed solution consisting of 1 mL of sample solution, 1 mL of 9 mmol/L salicylic acid–ethanol solution, 1 mL of 9 mmol/L FeSO_4_ and 1 mL of 8.8 mmol/L H_2_O_2_ was incubated at 37 °C for 30 min, after which the absorbance (A_1_) was recorded at 510 nm. An equal volume of 70% ethanol replaced the sample solution to prepare the blank (A_0_). In the reactive system, distilled water was used to replace H_2_O_2_ for the determination of absorbance A_2_. The analysis was conducted in triplicate. The ^·^OH scavenging rates of samples were calculated using the Formula (3):

(3) OH · scavenging rate %=1−A1−A2A0×100%

#### 3.4.4. Determination of ^·^O_2_^−^ Scavenging Ability

Scavenging rates of samples for ^·^O_2_^−^ were determined using the method previously reported by Giese [22], with some modifications. Then, 1 mL of sample solution and 5 mL of 50 mmol/L Tris-HCl Buffer reacted at 25 °C for 20 min, and then 1 mL 3 mmol/L of preheated pyrogallol was added, mixed and incubated at 25 °C for 5 min until termination of reaction by adding 1 mL of 10 mol/L HCl. The absorbance (A_1_) was determined at 320 nm, and a solution mixture without sample solution or pyrogallol reagent was used for the determination of absorbance values A_0_ and A_2_, respectively. Reduced vitamin C was used as a positive control. The analysis was conducted in triplicate. The determination consisted of three replicates. The ^·^O_2_^−^ scavenging rates of the samples were calculated using the Formula (4):

(4) O2− · scavenging rate %=1−A1−A2A0×100%

### 3.5. Statistical Analysis

Excel 2010 and SPSS 26.0 were used for the data processing analysis and standardization. Group comparisons were carried out using one-way ANOVA, and a *p*-value < 0.05 was regarded as significant. The mean ± standard deviation (SD) (*n* = 3) values were used to express all the results, which were collected in triplicate. Origin 2021 was used for the correlation analysis, principal component analysis (PCA), heat map production and visualization.

## 4. Discussion

### 4.1. Nutraceutical Analysis

There is no clear line between edible and non-edible flowers, as they are mainly separated according to whether they contain toxic substances. Edible flowers have been utilized as food for over 3000 years due to their great nutritional value, commonly in salads, sweets, beverages and sauces, and as vegetables in cooking and soups [23,24]. In Yunnan Province, flowers are usually used in the production of flower cakes, a local specialty food product. Based on edible experience and history, these 34 flowers used in this study were judged as edible flowers; thus, it is necessary to reveal their nutritional value and antioxidant activity. The nutritional value of flowers is greatly affected by the variety and the environment. Compared to that reported in the other literature, the 34 edible flowers used in this study generally had a high nutritional value. The moisture contents of flowers are closely related to flower blooming status and irrigation level and are usually used to calculate the dry matter content, which is the conversion basis of other nutritional indices [25]. Evidence showed that the moisture contents of most edible flowers ranged from 70 to 96.2%, e.g., Papilionaceae *Lathyrus odoratus* L., Rubiaceae *Pentas lanceolate* (Forssk.) K. Schum., Scrophulariaceae *Torenia fournieri* Linden. ex Fourn. and Begoniaceae *Begonia semperflorens cultorum* Hort. [4,26], which was in line with our result of 71.83~90.26% for the 34 edible flowers.

Flavonoid refers to a series of compounds formed by connecting two benzene rings with phenol hydroxyl group through the central three carbon atoms, and its basic parent nucleus is 2-phenylchromogen ketone [27]. There are many types of flavonoids in edible flowers, e.g., epicatechin, catechin, cyanidin, rutin and quercetin, that present in a wide range of plants with powerful antioxidant, anti-inflammatory, antitumor and blood-pressure and blood-sugar regulation properties; thus, they are commonly used as dietary supplements to promote health and illness prevention [2,13,27,28]. However, the nutritional and medicinal values of different flavonoids in flowers are not well characterized [29]. There are no recommended daily dietary amounts for different flavonoids, as well as flowers that are calculated based on the flavonoid content. The results of this experiment revealed that Asteraceae samples had high total flavonoid contents, and the largest value belonged to sample A3, at 8.53% (Table 1), which is significantly greater than 0.30% for Asteraceae *Calendula officinalis* L., 0.60% for Calycanthaceae *Chimonanthus praecox* (L.) Link, 7.15% for Oleaceae *Osmanthus fragrans* (Thunb.) Lour., 2.23% for Boraginaceae *Borago officinalis* L., 2.47% for Lamiaceae *Lavandula angustifolia* Mill. and 1.90% for Begoniaceae *B. semperflorens* Link et Otto [30,31]. It is also worth noting that the total flavonoid contents of Rosaceae samples varied greatly, with a range of 0.43~7.00%, suggesting that the variety was the key factor that affected flavonoid contents. Similar phenomena were found in the results of the assay of four rose cultivars described by Li [32].

Vitamin C plays vital roles in maintaining the normal growth and development of the human body and strengthening the resistance to diseases [33,34]. It can eliminate the superoxide, hydroxyl radicals and singlet oxygen via a reduction reaction and, thus, protects the organism against peroxidative damage. In addition, vitamin C is capable of helping to maintain many enzymes (monoxygenases and dioxygenases) in their required reduced forms [35]. Natural vitamin C from plants, e.g., fruits, vegetables and flowers, completely meets the needs of the body, with the recommended intake for adults being 70~90 mg/per day [36]. Studies founded that Rosaceae flowers in full bloom commonly had high contents of vitamin C, thus confirming our results that the vitamin C contents of all 10 Rosaceae flower samples were more than 63 mg/100 g, and half of them were greater than 100 mg/100 g (Table 1), making them significantly greater than the 29.84 mg/100 g of *B. officinalis*, 37.52 mg/100 g of *L. angustifolia*, 14.40 mg/100 g of Asteraceae *Bellis perennis* L. and 20.16 mg/100 g of *B. semperflorens* [31]. The vitamin C contents of the other three families, i.e., Asteraceae, Caryophyllaceae and Gentianaceae, are obviously less than this level. The high total sugar content is a key factor for flowers to become a more palatable edible material. Stefaniak [20] found that the contents of total soluble sugars and reducing sugars of five edible flowers (Phrymaceae *Mimulus × hybridus* L., Asphodelaceae *Hemerocallis × hybrida* Hort., Scrophulariaceae *Antirrhinum majus* L., Caryophyllaceae *Dianthus chinensis* L. and Lamiaceae *Monarda didyma* L.) ranged from 1.48 to 5.60% FW and 1.55 to 4.92% FW, respectively; these contents were obviously higher than the values determined in our work. However, for these 34 flowers, the protein contents in terms of total nitrogen were generally higher than those of edible flowers reported by Stefaniak [20] and Chensom [26]. Ash is a combination of various minerals and their oxides and basic phosphates. The total ash contents of these 34 flowers were generally above 5%, significantly higher than the contents of 0.53~1.56% that were reported for edible flowers [20], and this was speculated to be related to the high heavy metal soil background in planting areas.

### 4.2. Antioxidant Activity Analysis

The ability of natural materials to scavenge free radicals largely reflects their antioxidant capacity [37]. In fact, a single index lacks specificity and sensitivity and does not fully reflect the antioxidant capacity of flower samples. Four indices of FRAP values and scavenging rates of DPPH, ^·^OH and ^·^O_2_^−^ were used in this study to evaluate the antioxidant capacity of the 34 edible flowers. There were significant differences in the total antioxidant capacity among the 34 flowers, which were consistent with the results of 12, 23 and 65 edible flowers with the ranges of 0.82~70.42 mmol/100 g FW, 8.08~913.58 μmol/g FW and 4.17~362.02 mmol/100 g FW, respectively, evaluated by the FRAP assay [30,38,39]. According to the comprehensive comparison, the total antioxidant capacity of flowers from Rosaceae and Asteraceae was generally stronger than that of Caryophyllaceae and Gentianaceae flowers.

The results showed that these 34 samples had high DPPH scavenging rates, with a range of 6.32~87.98%, similar to the studies of Zeng [8]. The DPPH scavenging rates of their 19 edible flowers ranged from 8.12 to 94.24%. Compared with the ^·^OH scavenging rate range of 19 edible herbal flowers (33.51~93.22%), the range of these 34 flowers was much narrower, just 4.13~53.26%, particularly in flowers from the same family, but the average ^·^OH scavenging rates of the determined samples in our work were slightly lower than the values presented in the previous report (Figure 4). However, there is no detailed report on which family or genus of flowers has a higher ^·^OH scavenging rate. Our study provided a reference for the fact that the average ^·^OH scavenging rate of Rosaceae flowers was the largest, followed by Asteraceae and Caryophyllaceae, and the lowest was Gentianaceae. A similar pattern and trend also appeared in the ^·^O_2_^−^ scavenging capacity. The ^·^O_2_^−^ scavenging rates of the same family flowers were relatively close, and the average scavenging rates of 10 Rosaceae flowers were higher, followed by Caryophyllaceae and Asteraceae, and the lowest was Gentianaceae.

Since evaluating the quality of flowers by the content of a specific nutrient or the magnitude of a single antioxidant index alone will make the results lack scientific rigor and bias due to subjective factors, it is especially important to choose a suitable comprehensive quality evaluation method [40]. The PCA is a statistical analysis method that converts multiple indices into a limited number of parameters for a comprehensive evaluation, and it is widely used for the quality evaluation of fruits, vegetables and foods [39]. Jin completed a ranking of the comprehensive quality of tomatoes under different water-level deficit treatments, using the PCA method [40], while a cold-tolerant tomato was evaluated and screened by using the PCA in Cao’s study [41]. In this study, based on the nutrient content and antioxidant capacity of flowers, the top four results of the ranking of comprehensive quality of 34 flowers using the principal component analysis were R2 > R3 > R9 > R4 (Table 3). In addition, Pearson’s analysis showed that the contents of total flavonoids and vitamin C did not show a high causal correlation with the antioxidative properties of these flowers. It is speculated that the antioxidant process within plants is a result of complex multi-substance interactions, which need to be further studied from the perspective of metabolic pathways.

## 5. Conclusions

Our results indicated that the 34 edible flower samples from the families Rosaceae, Asteraceae, Caryophyllaceae and Gentianaceae had high a nutritional value and antioxidant capacity. However, there were significant differences in the nutrient contents and antioxidant properties of these 34 flowers. The comprehensive analysis, nutrient contents and antioxidant capacity of Rosaceae flower samples were generally better than those of the other three families. Taken together, these findings shed light on the potential health benefits of 34 edible flowers grown in Yunnan Province and could help guide the development of new functional foods and beauty products, as well as provide an additional source of flower food diversity to meet our growing health needs.

## Figures and Tables

**Table 1:** Nutrient contents of 34 flower samples distributed in 4 families, i.e., Rosaceae, Asteraceae, Caryophyllaceae and Gentianaceae.

Flower Samples		Content of Nutrient Composition						
		Moisture (%)	Total Flavonoids (%)	Vitamin C (mg/100 g)	Total Soluble Sugars (mg/100 g)	Reducing Sugars (%)	Ash (%)	Total Proteins (%)
Rosaceae	R1	75.78 ± 1.13 ^f^	6.61 ± 0.08 ^b^	143.80 ± 1.55 ^b^	108.50 ± 1.97 ^b^	0.34 ± 0.00 ^b^	5.13 ± 0.30 ^fg^	2.83 ± 0.14 ^g^
	R2	77.92 ± 0.73 ^e^	7.00 ± 0.21 ^a^	113.80 ± 0.62 ^e^	95.91 ± 0.47 ^c^	6.26 ± 0.16 ^a^	5.33 ± 0.12 ^ef^	4.68 ± 0.19 ^f^
	R3	71.83 ± 0.74 ^g^	2.16 ± 0.04 ^c^	127.60 ± 0.44 ^d^	34.20 ± 0.79 ^g^	0.10 ± 0.00 ^cd^	5.95 ± 0.26 ^ab^	3.28 ± 0.21 ^g^
	R4	86.45 ± 1.15 ^ab^	0.63 ± 0.01 ^fg^	63.11 ± 0.69 ^j^	27.03 ± 0.54 ^h^	0.12 ± 0.00 ^cd^	6.19 ± 0.18 ^a^	17.25 ± 0.50 ^c^
	R5	84.13 ± 0.98 ^cd^	0.74 ± 0.01 ^ef^	74.01 ± 0.07 ^i^	5.29 ± 0.70 ^i^	0.15 ± 0.00 ^c^	5.71 ± 0.15 ^bcd^	10.83 ± 0.35 ^e^
	R6	87.28 ± 0.51 ^ab^	0.61 ± 0.01 ^fg^	76.04 ± 0.15 ^h^	3.78 ± 0.45 ^i^	0.04 ± 0.00 ^d^	5.88 ± 0.08 ^abc^	20.72 ± 0.42 ^a^
	R7	88.02 ± 0.75 ^a^	0.43 ± 0.02 ^h^	132.40 ± 0.55 ^c^	68.15 ± 0.20 ^d^	0.33 ± 0.01 ^b^	5.05 ± 0.05 ^fg^	15.12 ± 0.55 ^d^
	R8	79.18 ± 1.00 ^e^	0.86 ± 0.02 ^e^	82.60 ± 0.03 ^g^	50.00 ± 0.62 ^f^	0.15 ± 0.00 ^c^	5.58 ± 0.21 ^cde^	18.80 ± 0.54 ^b^
	R9	82.65 ± 0.78 ^d^	1.67 ± 0.01 ^d^	162.20 ± 0.06 ^a^	112.60 ± 0.46 ^a^	0.32 ± 0.00 ^b^	4.91 ± 0.13 ^g^	10.84 ± 0.19 ^e^
	R10	85.85 ± 0.55 ^bc^	0.56 ± 0.03 ^gh^	93.13 ± 0.34 ^f^	56.42 ± 0.79 ^e^	0.20 ± 0.00 ^c^	5.51 ± 0.09 ^de^	18.61 ± 0.10 ^b^
Asteraceae	A1	88.13 ± 0.75 ^bc^	3.47 ± 0.05 ^d^	111.00 ± 0.82 ^a^	19.39 ± 1.71 ^f^	0.07 ± 0.00 ^ef^	7.56 ± 0.16 ^cd^	25.43 ± 0.48 ^a^
	A2	75.71 ± 0.41 ^h^	2.62 ± 0.05 ^f^	17.53 ± 0.29 ^f^	47.45 ± 1.38 ^b^	0.13 ± 0.00 ^e^	9.86 ± 0.33 ^a^	10.27 ± 0.37 ^cd^
	A3	82.58 ± 1.16 ^g^	8.53 ± 0.08 ^a^	38.52 ± 0.37 ^c^	13.98 ± 1.21 ^g^	0.06 ± 0.00 ^f^	7.78 ± 0.11 ^c^	19.97 ± 0.61 ^b^
	A4	84.97 ± 0.79 ^ef^	3.72 ± 0.15 ^d^	1.62 ± 0.06 ^i^	32.43 ± 0.85 ^de^	5.46 ± 0.05 ^a^	6.40 ± 0.20 ^b^	7.58 ± 0.27 ^e^
	A5	83.78 ± 0.87 ^fg^	2.70 ± 0.02 ^f^	61.25 ± 0.72 ^b^	19.69 ± 0.58 ^f^	3.71 ± 0.00 ^b^	9.00 ± 0.12 ^e^	19.37 ± 0.29 ^b^
	A6	87.04 ± 0.43 ^cd^	8.36 ± 0.26 ^ab^	3.90 ± 0.30 ^h^	35.06 ± 0.42 ^cd^	3.03 ± 0.05 ^c^	7.67 ± 0.03 ^cd^	10.98 ± 0.58 ^c^
	A7	85.01 ± 0.58 ^ef^	8.36 ± 0.01 ^ab^	24.60 ± 0.24 ^e^	31.97 ± 1.34 ^de^	0.06 ± 0.00 ^f^	7.71 ± 0.21 ^cd^	9.43 ± 0.39 ^d^
	A8	90.26 ± 0.69 ^a^	4.77 ± 0.04 ^c^	26.69 ± 0.08 ^d^	37.17 ± 2.41 ^c^	0.11 ± 0.00 ^ef^	8.78 ± 0.18 ^b^	19.27 ± 0.20 ^b^
	A9	89.65 ± 0.52 ^ab^	8.06 ± 0.14 ^b^	17.74 ± 0.01 ^f^	29.86 ± 1.75 ^e^	0.08 ± 0.00 ^ef^	8.66 ± 0.06 ^b^	24.18 ± 0.99 ^a^
	A10	86.00 ± 0.85 ^de^	3.58 ± 0.15 ^d^	8.86 ± 0.16 ^g^	66.76 ± 2.45 ^a^	0.20 ± 0.00 ^d^	7.31 ± 0.25 ^d^	9.19 ± 0.64 ^d^
Caryophyllaceae	C1	84.87 ± 0.03 ^b^	0.99 ± 0.00 ^c^	10.90 ± 0.28 ^b^	3.83 ± 0.41 ^g^	1.43 ± 0.00 ^d^	6.39 ± 0.06 ^b^	14.67 ± 0.55 ^c^
	C2	86.51 ± 0.00 ^ab^	0.40 ± 0.00 ^f^	17.33 ± 0.80 ^a^	31.43 ± 0.28 ^b^	0.84 ± 0.00 ^f^	3.57 ± 0.17 ^e^	13.87 ± 0.54 ^c^
	C3	84.64 ± 0.01 ^b^	0.69 ± 0.00 ^d^	6.91 ± 0.34 ^de^	11.13 ± 0.32 ^e^	0.08 ± 0.00 ^h^	6.16 ± 0.11 ^b^	18.24 ± 0.58 ^b^
	C4	88.95 ± 0.00 ^a^	0.54 ± 0.00 ^e^	6.12 ± 0.14 ^b^	19.98 ± 0.80 ^c^	1.44 ± 0.00 ^d^	6.12 ± 0.14 ^b^	13.59 ± 0.71 ^cd^
	C5	86.94 ± 0.01 ^ab^	1.32 ± 0.00 ^b^	3.55 ± 0.12 ^e^	50.93 ± 0.84 ^a^	2.01 ± 0.00 ^b^	3.55 ± 0.12 ^e^	10.51 ± 0.19 ^e^
	C6	86.18 ± 0.01 ^b^	0.35 ± 0.00 ^f^	5.42 ± 0.16 ^c^	50.48 ± 0.14 ^a^	1.08 ± 0.00 ^e^	5.42 ± 0.16 ^c^	18.05 ± 0.81 ^b^
	C7	75.37 ± 0.00 ^d^	0.66 ± 0.00 ^d^	4.59 ± 0.21 ^d^	18.67 ± 0.69 ^c^	0.30 ± 0.00 ^g^	4.59 ± 0.21 ^d^	19.55 ± 1.14 ^ab^
	C8	85.93 ± 0.01 ^b^	0.36 ± 0.00 ^f^	6.25 ± 0.02 ^b^	19.98 ± 0.98 ^c^	1.58 ± 0.00 ^c^	6.25 ± 0.05 ^b^	11.85 ± 0.33 ^de^
	C9	86.52 ± 0.01 ^ab^	1.61 ± 0.00 ^a^	7.54 ± 0.18 ^a^	7.730 ± 0.56 ^f^	3.77 ± 0.00 ^a^	7.54 ± 0.18 ^a^	20.85 ± 0.80 ^a^
	C10	78.62 ± 0.01 ^c^	1.59 ± 0.00 ^a^	7.86 ± 0.26 ^a^	13.58 ± 0.21 ^d^	1.52 ± 0.00 ^cd^	7.86 ± 0.26 ^a^	8.260 ± 0.85 ^f^
Gentianaceae	G1	80.74 ± 0.01 ^a^	0.62 ± 0.00 ^b^	5.28 ± 0.06 ^ab^	27.06 ± 0.66 ^b^	2.10 ± 0.00 ^b^	5.28 ± 0.06 ^ab^	16.61 ± 0.35 ^a^
	G2	77.13 ± 0.00 ^b^	3.24 ± 0.00 ^a^	5.11 ± 0.14 ^b^	11.64 ± 0.35 ^c^	1.20 ± 0.00 ^c^	5.11 ± 0.14 ^b^	2.79 ± 0.04 ^d^
	G3	77.91 ± 0.00 ^b^	0.51 ± 0.00 ^c^	5.65 ± 0.25 ^ab^	40.49 ± 0.77 ^a^	7.82 ± 0.00 ^a^	5.65 ± 0.25 ^ab^	11.79 ± 0.54 ^b^
	G4	82.18 ± 0.02 ^a^	0.53 ± 0.00 ^bc^	5.82 ± 0.37 ^a^	40.68 ± 1.34 ^a^	0.09 ± 0.00 ^d^	5.82 ± 0.37 ^a^	9.79 ± 0.34 ^c^

Values are shown as the mean ± SD (*n* = 3). Different superscript lowercase letters indicate statistically significant differences in the content of the same nutrient among flower samples in the same family, *p* < 0.05.

**Table 2:** Eigenvalues and cumulative contribution of each principal component to the nutritional quality and antioxidant capacity of the 34 edible flower samples.

Principal Component (PC)	Eigenvalues (λ)	Variance (%)	Cumulative (%)
PC1	3.18	45.45	45.45
PC2	1.47	20.98	66.43
PC3	1.04	14.84	81.27

**Table 3:** Comprehensive quality score and ranking of the 34 edible flower samples.

Samples	F1		F2		F3		Comprehensive Evaluation	
	Sore	Rank	Sore	Rank	Sore	Rank	Sore	Rank
R1	29.30	4	−0.03	14	−0.40	21	16.33	4
R2	41.66	1	5.41	2	2.50	2	25.19	1
R3	41.36	2	0.77	11	0.04	16	23.37	2
R4	22.97	5	−2.10	29	0.31	11	12.37	5
R5	17.97	7	−1.16	20	−0.18	19	9.73	7
R6	21.15	6	−2.59	30	0.18	13	11.20	6
R7	13.67	10	−2.84	32	−0.84	29	6.76	11
R8	14.05	9	−1.89	28	−0.52	25	7.28	9
R9	31.01	3	−1.77	26	−0.37	20	16.84	3
R10	15.12	8	0.13	13	−0.77	28	8.36	8
A1	13.46	11	−0.51	16	−0.97	31	7.23	10
A2	−9.16	16	−0.42	15	−0.41	22	−5.31	17
A3	−4.83	14	3.17	6	−1.69	32	−2.18	14
A4	−17.74	34	4.11	4	1.37	3	−8.62	33
A5	1.81	12	2.75	7	0.40	9	1.80	12
A6	−8.94	15	5.67	1	0.16	14	−3.50	15
A7	−12.39	22	4.87	3	−2.16	34	−6.06	19
A8	−2.89	13	1.32	9	−0.88	30	−1.43	13
A9	−10.63	19	3.96	5	−1.82	33	−5.25	16
A10	−14.24	31	−0.54	17	−0.67	27	−8.24	29
C1	−10.62	18	−5.13	34	1.05	5	−7.09	23
C2	−13.10	24	−0.93	18	−0.08	17	−7.60	25
C3	−13.95	28	−2.89	33	−0.15	18	−8.59	32
C4	−15.70	33	−1.42	24	0.24	12	−9.12	34
C5	−11.22	21	−2.70	31	0.95	6	−6.81	22
C6	−13.94	27	−1.81	27	0.13	15	−8.26	30
C7	−10.48	17	−1.39	23	−0.50	24	−6.32	20
C8	−14.59	32	−1.56	25	0.50	8	−8.48	31
C9	−13.76	26	0.85	10	1.13	4	−7.28	24
C10	−14.16	30	−1.02	19	0.38	10	−8.13	28
G1	−13.50	25	−1.37	22	0.64	7	−7.80	27
G2	−10.91	20	0.51	12	−0.42	23	−6.05	18
G3	−14.03	29	1.92	8	3.37	1	−6.75	21
G4	−12.78	23	−1.36	21	−0.53	26	−7.60	25

## Data Availability

All of the data are contained in the article.
